# Protective Effects of *Ammannia baccifera* Against CCl_4_-Induced Oxidative Stress in Rats

**DOI:** 10.3390/ijerph16081440

**Published:** 2019-04-23

**Authors:** Lavanya Goodla, Manjunath Manubolu, Kavitha Pathakoti, Thanasekaran Jayakumar, Jeon-Rong Sheu, Mike Fraker, Paul B. Tchounwou, Parthasarathy R. Poondamalli

**Affiliations:** 1 Department of Biochemistry, Sri Venkateswara University, Tirupati 517502, India; prparthasarathy.svu@gmail.com; 2 Aquatic Ecology Laboratory, Department of Evolution, Ecology and Organismal Biology, The Ohio State University, Columbus, OH 43212, USA; manubolu.1@osu.edu (M.M.); mfraker2@gmail.com (M.F.); 3 Department of Biology, Jackson State University, Jackson, MS 39217, USA; kavitha.pathakoti@jsums.edu (K.P.); paul.b.tchounwou@jsums.edu (P.B.T.); 4 Graduate Institute of Medical Sciences, College of Medicine, Taipei Medical University, Taipei 110, Taiwan; tjaya_2002@yahoo.co.in (T.J.); sheujr@tmu.edu.tw (J.-R.S.)

**Keywords:** antioxidant enzymes, CCl_4_, gene expression, hepato-protection, iso-enzyme, oxidative stress, phytochemicals, UHPLC-QTOF-MS

## Abstract

*Ammannia baccifera* Linn. is commonly used as a traditional medicine in India and China. The antioxidant potential of an ethanolic extract of *A. baccifera* (EEAB; 250 mg/kg and 500 mg/kg) was evaluated against CCL_4_-induced toxicity in rats. Antioxidant activity was assessed by measuring the enzymatic and non-enzymatic antioxidants. Phytochemical constituents of EEAB were also analyzed by using UHPLC-QTOF-MS. EEAB treatment markedly reduced CCl_4_ effects on lipid peroxidation, cholesterol, triacylglycerides, and protein carbonyls. It increased the levels of phospholipids, total sulfhydryl, and antioxidant enzymes, which were reduced by CCl_4_ intoxication. Treatment with EEAB significantly alleviated the CCl_4_ effect on non-enzymatic antioxidants. Isoenzyme pattern analyses revealed that significant alterations in superoxide dismutase (SOD1), glutathione peroxidase (GPx2, GPx3), and catalase (CAT) occurred in rats that were exposed to CCl_4_ and restored post EEAB treatment. Moreover, CCl_4_-induced down regulation of SOD, CAT, and GPx gene expression was conversely counteracted by EEAB. Its bioactivity may be due to its incorporation of major compounds, such as chlorogenic acid, quercetin, protocatechuic acid, lamioside, crocetin, and khayasin C. These results suggest that EEAB may be used as a potent antioxidant and hepatoprotective agent since it is a rich source of flavonoids and phenolic compounds.

## 1. Introduction

Carbon tetrachloride (CCl_4_) is a classical hepatotoxicant model that is often studied for xenobiotic-induced oxidative hepatotoxicity [1]. Bio-activation of the hepatic cytochrome P_450_ system catabolizes CCl_4_ to trichloromethyl radical (CCl_3_^−^), which is a highly reactive metabolite. This CCl_3_^−^ radical further reacts with oxygen to form the most toxic reactive trichloromethyl peroxy radical, which can bind to macromolecules. This leads to cell membrane disruption and cell death [2]. Oxidative stress arises when there is an imbalance between production and scavenging of reactive oxygen species (ROS). The major cause for various degenerative diseases including some hepatopathies is oxidative stress [3]. Natural antioxidants are considered to be effective and safe alternative treatments for hepatotoxicity in comparison to synthetic antioxidants, which may be carcinogenic [4,5].

Worldwide, about 60% of the population uses traditional medicines. During the last few decades, there has been a remarkable development in the field of herbal medicine [6]. *Ammannia baccifera* Linn. (Family: Lythraceae) is being used as traditional medicine in India and China. It has a wide range of therapeutic and pharmacological properties, including antiurolithiasis, antibacterial, central nervous system (CNS) anti-depressant, antifungal, antitumor, anti-inflammatory, and antisteroidogenic activities [7,8,9]. Our earlier in vitro study described the antioxidant potential of the ethanolic extract of *Ammannia baccifera* (EEAB) [10]. Most of the available reports on the beneficial effects of *A. baccifera* extract showed that secondary metabolites of its extract, which are of a phenolic nature, are responsible for the array of biological activities. No clear molecular mechanism of action has been revealed to date.

Ultra-high performance liquid chromatography (UHPLC) with mass spectrometry (MS), which facilitates rapid separation and identification of components of the plant extracts [11], has been widely used for metabolic profiling. The use of high resolution mass spectrometry (HRMS) further improves sensitivity and selectivity, which has aided concurrent identification of numerous known and unknown metabolites [12]. Despite *Ammannia baccifera* having long been used as traditional medicine in India and China, there is no detailed information on the antioxidant activity of this herbal plant in vivo. Therefore, it is useful to examine the hepatoprotective effects of EEAB in rats after CCl_4_ stress. The isoenzyme composition and gene expression of enzymes—CuZn-SOD, CAT, and GPx of liver were also studied, along with chemical characterization of EEAB through ultra-high performance liquid chromatography coupled with quadrupole time-of-flight mass spectrometry (UHPLC-QTOF-MS).

## 2. Materials and Methods

### 2.1. Plant Material

Whole herbs of *Ammannia baccifera* were procured from the local areas of Tirumala Tirupati Hills, Andhra Pradesh, India. The herb was identified and authenticated by the Botanist, Dr. K. Madhava Chetty, Sri Venkateswara University, India.

### 2.2. Preparation of A. baccifera Extract

The whole herbs of *A. baccifera* (15–20 plants) were shade dried and powdered using a blender (Hammer Mill) and 100 g of this powder was soaked in 500 mL ethanol (95%) for a week in dark conditions and at room temperature (26–28 °C). After a week, the extract was concentrated after filtration in a rotary evaporator and stored at −20 °C. The yield of the extract was 8.6% (~9 g from 100 g of dried powder).

### 2.3. Phytochemical Analysis by UHPLC-QTOF-MS

Analyses were done in an Agilent 1290 UHPLC system (Agilent Technologies Inc., Palo Alto, CA) comprising a 1260 infinity Nano HPLC with Chipcube, 6550 iFunnel Q-TOFs. Full scans from *m*/*z* 50–1000 @ 1.2 scans/s were collected. A Rapid Resolution HD column -Zorbax SB C-18 with 2.1 mm × 100 mm × 1.8µm dimensions was used at 35 °C with a flow rate of 0.4 mL/min and an injection volume of 20 µL. The other conditions used: Ionization: Heated Electrospray (HESI). Transfer line temperature: 350 °C. Spray voltage: 4 kV. Nebulizing gas: Nitrogen generated by Peak Scientific NM32LA model nitrogen generator. Analysis in a positive mode was carried out in a gradient mobile phase with binary solvents containing water with 0.1% formic acid as mobile phase A and Methanol with 0.1% formic acid as mobile phase B. The mobile phase B varied from 0–95% from 0–50 min, 95% B from 50–55 min, and initial conditions from 55.1–60 min. A similar program was used in a negative ionization mode with mobile phase A, as water and mobile phase B, as acetonitrile. The maximal tolerated *m*/*z* deviation was set to 10 ppm [13]. The molecular formulas of compounds were identified by HRMS by comparing theoretical and observed mass. Compounds were identified by comparing the mass values with the existing databases like the knapsack family databases, Metlin, and Lipidmaps that were identified from Mass Hunter Qualitative analysis software (version B.06.00) Agilent Technologies, Santa Clara, CA, USA.

### 2.4. Animals

Male rats (Albino Wistar, 200 to 250 g) were purchased from the local supplier and acclimatized for one week prior to experimentation. They had free access to a pelleted diet (Hindustan Lever Ltd., Mumbai, India) and water *ad libitum* at 25 ± 2 °C with a 12-h light/dark cycle. Female rats were excluded in this study to avoid the data variability caused by hormonal cycles in females. All the procedures were in accordance with the Institutional regulations, controlled by the committee CPCSEA of Ministry of Social Justice and Empowerment of India. Approved by the Institutional Animal Ethics Committee [(Regd.No. 438/01/a/CPCSEA/dt 17.7.2001) in its resolution number 9/IAEC/ SVU/ Zool, dated 4.3.2002].

### 2.5. Experimental Procedure

According to previous acute toxicological reports of EEAB, 2000 mg/kg body weight of EEAB was proven to be nontoxic to rats [14]. Earlier sub-acute studies that used four different doses of EEAB (50, 100, 250, and 500 mg/Kg body weight) to determine the dose-dependent effects in rats revealed no signs of toxicity [14]. Hence, we chose the 250 and 500 mg/kg doses of EEAB in the present study.

Animals were randomly assigned into four groups of six rats in each experimental group and EEAB dissolved in distilled water was administered daily via intra-gastric intubation.

Group I: control − olive oil (1 mL/kg)
Group II: toxic control − 30% CCl_4_
Group III: EEAB 250 mg/kg + 30% CCl_4_
Group IV: EEAB 500 mg/kg + 30% CCl_4_

Excluding the control (Group I), all animals in Groups II, III, and IV received 30% CCl_4_ olive oil (1 mL/Kg) via intraperitoneal injection for one time every three days for an eight-week period [15]. Animals in Groups III and IV, respectively, received 250 mg/kg and 500 mg/kg EEAB, 72 h prior to CCl_4_ treatment.

After eight weeks, experimental rats were anesthetized and sacrificed by cervical dislocation, and the liver samples were collected, rinsed with saline solution, and stored at −80 °C. Samples were homogenized as described earlier [16]. The protein was measured by the Bradford [17] method.

### 2.6. Quantification of Lipid Peroxidation (LPO) and Analysis of Lipids

Lipid Peroxidation (LPO) was measured by using malondialdehyde (MDA) as the standard, where thiobarbituric acid reacts with MDA forming a pink colored complex, due to peroxidation of lipids, which is measured at 532 nm [18]. Chloroform-methanol mixture (2:1 *v*/*v*) [19] was used for extraction of lipids from liver tissue to measure the amount of total cholesterol [20], triacylglycerides [21,22], and phospholipids [23].

The cholesterol content was measured using FeCl_3_-uranyl acetate and H_2_SO_4_-FeSO_4_ as reagents. After 20 min of incubation, the purple color developed was read at 540 nm. For triacylglyceride measurement, chloroform-methanol mixture (2:1) and activated silicic acid was added to the lipid extract for phospholipid absorption followed by saponification and incubation with sulphuric acid, sodium meta periodate, and chromotropic acid. After incubation, thiourea solution was added to the final contents and the absorbance was read at 570 nm. Phospholipids were measured using the acid digestion process using perchloric acid and the absorbance was measured at 710 nm, after the addition of ascorbic acid and ammonium molybdate.

### 2.7. Analysis of Protein Damage: Estimation of Protein Carbonyl and Total Sulfhydryl (Thiol) Contents

The protein carbonyl content of liver homogenates was measured using 2,4-Dinitrophenylhydrazine, which is measured at 370 nm [24]. Briefly, 100 µL of sample was incubated with 100 µL of DNPH (100 mM) and the protein was precipitated with 20% TCA. The resultant pellet was dissolved in 6 µM guanidine hydrochloride (pH 6.5), after washing with ethyl acetate:ethanol (1:1 *v*/*v*).

Estimation of total sulphydryl content was based on 5,5′-dithiobis-(2-nitrobenzoic acid) (DTNB) reaction, where DTNB is reduced by the thiol group to form 1 mole of 2-nitro-5-mercaptobenzoic acid per mole-SH that was read at 412 nm [25].

### 2.8. Antioxidants

Standard methods were used for estimating the Superoxide dismutase (SOD), Catalase (CAT), and Glutathione peroxidase (GPx), as described in the earlier study [16]. SOD was quantified according to the method based on the oxidation of epinephrine transition by the enzyme to form an adenochrome, which was measured at 470 nm (1 unit of SOD = Enzyme required to inhibit 50% of epinephrine auto-oxidation) [26]. Quantification of CAT activity was measured based on the decomposition of H_2_O_2_ by the enzyme and the decrease in absorbance at 240 nm was used to determine the activity (μmol of H_2_O_2_ consumed/min/mg protein) [27]. GPx activity was determined by continuous monitoring of NADPH oxidation in the reaction of cumene hydroperoxide decomposition [28].

The non-enzymatic antioxidants, such as reduced glutathione (GSH), vitamin C, and vitamin E were assessed by the standard methods. GSH was determined by the reaction with 5,5′-dithiobis-2 nitrobenzoic acid, which forms a yellow-colored complex and was read at 412 nm [29]. Vitamin C was analyzed by the oxidative reaction with copper to form dehydroascorbic acid, which reacts with 2,4-dinitrophenyl hydrazine to produce a product with an absorption maximum at 520 nm [30]. Vitamin E was quantified based on the Emmeric—Engel reaction [31].

### 2.9. Separation and Quantification of Isozyme Response by Electrophoresis

Following the standard method [32], gel electrophoresis with non-denaturing polyacrylamide was performed and the SOD, CAT, and GPx activity staining methods were followed, as described earlier [16]. The SOD isozymes were determined by soaking the gel in a 50-mm Tris-HCl buffer reagent comprised of nitro-blue tetrazolium (NBT), ethylene diamine tetra acetic acid, and riboflavin and incubated in dark for 30 min. CAT isozymes were detected by soaking the gel for 10 min in a 5-mm H_2_O_2_ solution, rinsed using double distilled water, and then stained with a reaction mixture containing 1% of ferric chloride and potassium ferricyanide (*w*/*v*). GPx isozymes were detected by immersing the gel in 50-mm Tris-HCl buffer containing NBT and phenazine methosulphate. Densitometric scanning of stained gels was performed to quantify the band areas of isozymes.

### 2.10. RNA Extraction and Quantification by a Reverse Transcriptase-Polymerase Chain Reaction (RT-PCR)

Total RNA was extracted following a prescribed kit protocol [33], and gene expression (SOD, CAT and GPX) was measured using the RT-PCR method, as previously described [16]. The thermal cycler (Eppendorf Mastercycler ep gradient S, motorized lid, 200–240 V) was used to complete the RT-PCR reaction under the following conditions. One reverse transcription step for 30 min at 50 °C. The initial enzyme activation step of 15 min at 95 °C. An amplification step for 30 cycles. The initial denaturation step for 30 s at 94 °C. An annealing step for 30 s at 58 °C and an extension step for 1 min at 72 °C [34]. The gene-specific primers used are presented in Table 1. The RT-PCR product (10 µL) was resolved on agarose gel (2%) to compare the quantity of steady state mRNA. Each target gene expression was standardized with β-actin (internal control gene) and represented as a ratio.

### 2.11. Statistical Analysis

The analysis was carried out using one-way Analysis of Variance (ANOVA), which was followed by the Duncan’s Multiple Range Test (DMRT). The data was tested for normality and homoscedascity using the SPSS software. Statistical Package for Social Sciences (SPSS) 15.0 (IBM Corporation, Chicago, IL, USA) was used for analysis and the *p*-value was set at *p* < 0.05. Data are presented as means ± SEs.

## 3. Results

### 3.1. Identification of Metabolites by UHPLC-QTOF-MS

The total chromatogram for the positive ion is presented in Figure S1. The results show that the positive ion chromatogram yielded significantly higher signals. In the negative mode, none of the compounds were identified (data not presented). The putative identities of 16 are identified in a positive mode and are summarized in Table 2. The compounds identified are hydroxycinnamic acids, flavonoids, terpenoids, and fatty acids. The major compounds include chlorogenic acid, quercetin, pentahydroxy flavanone, cosmosiin, dihydromyricetin, and protocatechuic acid, and terpenoids including lamioside, crocetin, and khayasin C.

### 3.2. Lipid Peroxidation and Lipid Profile

The hepatic LPO levels including cholesterol, triacylglycerides, and phospholipids are presented in Table 3. A marked rise in hepatic MDA, cholesterol, and triacylglycerides was noticed in CCl_4_-treated rats. Hepatic LPO, cholesterol, and triacylglycerides levels returned to normal after treatment with ethanolic extract of *A. baccifera* (EEAB). CCl_4_ decreased the hepatic phospholipid levels considerably, which were restored towards the control level by using EEAB.

### 3.3. Total Protein, Protein Carbonyl, and Total Sulfhydryl Content

Total protein levels were significantly depleted in CCl_4_-intoxicated rats. Treatment with EEAB at two doses significantly increased the protein content of liver tissue, when compared to rats treated with CCl_4_ alone (Table 4). The hepatic protein carbonyl levels were considerably augmented in CCl_4_ exposed rats. Administration of EEAB caused a substantial reduction (*p* < 0.01) in the levels of protein carbonyls of the liver tissue when compared to rats treated with CCl_4_ alone (Table 4).

The levels of total sulfhydryl groups are presented in Table 4. CCl_4_-intoxicated rats (Group II) showed a significant reduction in the levels of hepatic total sulfhydryl’s in comparison to the control (Group I). These variations were restored toward normal levels in both groups (Group III and Group IV) of EEAB-treated rats (Table 4).

### 3.4. Spectrophotometric Analysis of Antioxidants

The hepatic antioxidant and non-enzymatic antioxidants are shown in Table 5. Significant depletion of all the studied antioxidants was observed in liver tissues of CCl_4_-treated rats. The levels of all these enzymatic and non-enzymatic antioxidants were re-established toward control levels after treatment with EEAB.

### 3.5. Expression of Antioxidant Enzyme Proteins

**Figure 1:**
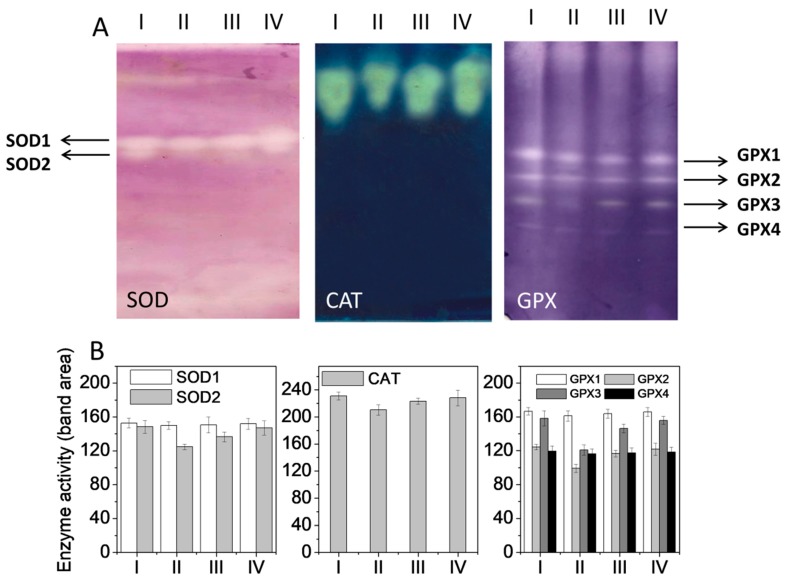
Effect of EEAB on the activity of antioxidant enzymes in the liver of CCl_4_-treated rats. (**A**) Electrophoretic pattern of antioxidant enzymes. (**B**) Densitometric analysis of antioxidant enzymes. Treatments—I: control. II: CCl_4_ 30%. III: EEAB (250 mg/kg) + CCl_4_ 30%. IV: EEAB (500 mg/kg) + CCl_4_ 30%. Values are mean ± SEM (*n* = 2).

Electrophoretic densitometric patterns of SOD, CAT, and GPx isozymes in all four treatment groups are represented in Figure 1A,B. Two isozymes of SOD (SOD1 and SOD2) were observed in the liver tissue (Figure 1A). The zymogram shows that the differences in band areas of isozyme SOD1 were basically comparable in all the groups while the SOD2 isoform showed decreased staining intensity in the CCl_4_ group, in contrast with the control. The staining intensity was more intense for SOD_2_ in EEAB-treated Group IV, in comparison to EEAB-treated Group III and CCl4-intoxicated Group II (Figure 1A,B).

The CAT isozyme activity staining zymogram is shown in Figure 1A. The CAT isozyme electrophoretic pattern showed a single band with minor changes in the band area. The staining intensity and band area of the CAT isozyme was significantly reduced in CCl_4_-intoxicated rats, in comparison with the control. EEAB treatment at two doses displayed a substantial increase in the staining intensity and band area of the CAT isozyme, compared to CCl_4_-intoxicated rats (Figure 1B).

Figure 1A depicts the GPx isozymes and the activity-staining zymogram. GPx activity bands revealed four isozymes: GPx1, GPx2, GPx3, and GPx4. Isozymes GPx1 and GPx4 were considered to be bands that were principally comparable in all the groups (Figure 1B). However, a noticeable diminution in the band areas of the enzymes GPx2 and GPx3 was seen in the group treated with CCl_4_ (Group II), in contrast with the control (Group I). EEAB treatment (Group III and IV) revealed enhanced band areas for the isoenzymes GPx2 and GPx3 in comparison with the Group II rats intoxicated with CCl_4_.

### 3.6. Antioxidant Enzymes: mRNA Expression

**Figure 2:**
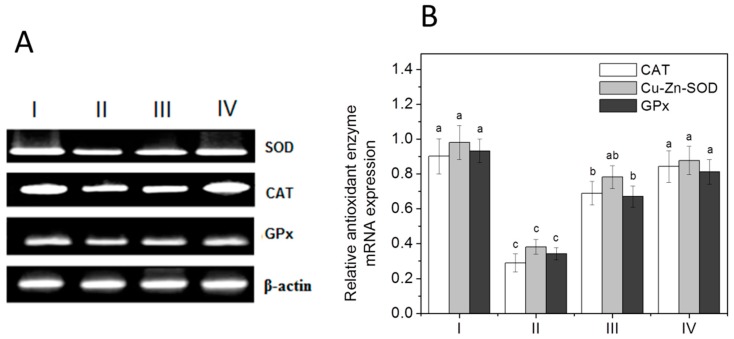
Effect of EEAB on mRNA expression of antioxidant enzymes in the liver of CCl_4_-treated rats. (**A**) RT-PCR profile. (**B**) Histogram of relative transcript levels. Treatments—I: control. II: CCl_4_ 30%. III: EEAB (250 mg/kg) + CCl_4_ 30%. IV: EEAB (500 mg/kg) + CCl_4_ 30%. Values are mean ± SEM (*n* = 3). Means with different suffix letters (a, b and c) differ significantly (*p* < 0.05).

To further confirm the observed changes in the spectral analysis and isozyme pattern of antioxidant enzymes, RT-PCR was used to examine the relative gene expression profiles of antioxidants in the liver with primers that are gene specific, as shown in Table 1. For confirming the enzymatic antioxidant alterations at the gene transcription level with the EEAB treatment, the measurement of the ratio of SOD/catalase/GPx mRNA to β-actin mRNA was done. The electrophoretic profile of the RT-PCR products of SOD, CAT, and GPx along with the housekeeping gene (β-actin) is represented in Figure 2A. The relative mRNA expression levels of CAT (32%), GPx (36.5%), and SOD (37%) antioxidant genes were noticeably downregulated in the CCl_4_-intoxicated group (Figure 2B). Notable upregulation in SOD (43%, 52.3%), CAT (44.4%, 61.4%), and GPx (35.3%, 51%) mRNA levels were observed in both the doses of EEAB-treated groups correspondingly, compared to Group II rats intoxicated with CCl_4_.

## 4. Discussion

CCl_4_, which is a strong hepatotoxic agent, has been widely used to establish animal models for screening the hepatoprotective activities of drugs [16,35]. Oxidative stress plays a vital role in CCl_4_-induced toxicity. CCl_4_ is converted to a trichloromethyl-free radical by cytochrome P_450_, which begins a series of free radical reactions leading to an increase in LPO. This disturbs the membrane integrity and Ca^2+^ homeostasis to produce hepatocellular damage [36]. The use of phytochemicals with antioxidant activity can offer protection against the oxidative damage [37]. The present investigation revealed that an ethanolic extract of *A. baccifera* (EEAB) significantly attenuated the CCl_4_ induced oxidative stress by augmenting the endogenous antioxidant levels.

Although qualitative screening of *A. baccifera* chemical compounds has been reported earlier using different analytical techniques [38], in this study, we used UHPLC-QTOF-MS, which significantly facilitates the simultaneous detection of compounds in lower amounts [39]. The major phytochemicals identified in the present study include chlorogenic acid, quercetin, pentahydroxy flavanone, cosmosiin, dihydromyricetin, protocatechuic acid, lamioside, crocetin, and khayasin, which belong to the group of polyphenolic compounds. The antioxidant and hepatoprotective activity of EEAB may be credited to the presence of these phytochemicals. Flavonoids and polyphenolic compounds are well-known natural antioxidants and these compounds counteract oxidative stress by direct ROS scavenging activity, metal chelation, and induction of antioxidant enzymes as well as phase II detoxifying enzymes [40]. Hepato-protective and antioxidant activities of chlorogenic acid, quercetin, cosmosiin, and protocatechuic acid have been well recognized [41,42,43,44]. Dihydromyricetin is a natural antioxidant and a potent depigment agent [45].

The mechanism of CCl_4_-induced liver injury is the stimulation of LPO and generation of ROS, which was reported earlier [36]. In this study, significantly higher MDA, an end-product of membrane LPO, was observed in CCl_4_-treated rats indicating hepatic damage with a concomitant decrease in antioxidant levels. Treatment with EEAB prevented the oxidative damage by reducing LPO and restoring the antioxidant levels when compared to the control. This could be attributed to the existence of radical scavenging antioxidant chemical constituents, as identified from UHPLC-QTOF-MS. Augmentation of lipids is considered to be a pathological state, which is an indication of impaired liver function under chronic accumulation [46]. An increased cholesterol and triacylglycerides level was observed after CCl_4_ intoxication. Earlier studies showed that the CCl_4_ causes the synthesis of cholesterol to rise in hepatocytes [2]. Decreased levels of phospholipids were observed after CCl_4_ treatment, which could be due to an augmentation in phospholipase activity [47,48], as phospholipase causes the cell membrane damage degrading membrane phospholipids [48]. Furthermore, these phospholipids are more vulnerable to CCl_4_-induced LPO than other lipid classes [49] and play a central role in the transport of triacylglycerides. During regular lipoprotein metabolism, phospholipids are broadly converted into triacylglycerides [50] (Wiggins and Gibbons, 1996). Furthermore, EEAB treatment normalized all the abnormal parameters due to CCl_4_ intoxication to near control levels. The present results are in accordance with previous studies related to lipid profiles after curcumin treatment against CCl_4_ toxicity [51].

CCl_4_ caused a significant augmentation in protein carbonyl contents and decreased total sulfhydryl levels. This is an indication of protein damage and increased levels of protein carbonyls causing changes in protein conformations, which leads to enhanced aggregation, fragmentation, distortion of secondary and tertiary structure, and vulnerability to proteolysis and diminution of normal function [52]. Thus, conservation of the protein redox status is vital for cell function. Any modifications in protein structure may lead to hepatotoxicity at the molecular level. Our present findings are in accordance with other studies showing an increased level of protein carbonyl contents after CCl_4_ treatment [53]. Co-treatment with EEAB reduced the protein carbonyl contents and restored the sulfhydryl levels toward the level of the control group.

Reduced Glutathione (GSH) is the key non-enzymatic antioxidant that regulates the intracellular redox homeostasis, which is ubiquitously present in all cell types to protect from deleterious effects of ROS [54]. CCl_4_ significantly reduced the GSH levels. The reestablished GSH levels, after EEAB treatment, could be due to regeneration and synthesis of GSH. The increased GSH levels can protect the liver from oxidative damage by directly scavenging the ROS, or being a component of the GSH redox system, which includes GPx, glutathione reductase, and glutathione-s-transferase (GST) [55]. Furthermore, we found a marked decrease in vitamin C and vitamin E after CCl_4_ treatment, which were recovered by EEAB treatment. These findings are in good agreement with the earlier studies [56]. The increased level of vitamin C and vitamin E in EEAB-treated rats reveals the anti-oxidative nature of the plant extract. The extract may scavenge the free radicals and, thus, maintain the normal level of vitamin C and vitamin E. It is well established that GSH in blood keeps up the cellular levels of the active forms of vitamin C and vitamin E by neutralizing the free radicals. When there is a reduction in the GSH content, the cellular levels of vitamin C are also lowered, which indicates that GSH, vitamin C, and vitamin E are closely interlinked to each other [57]. Reduction in cellular GSH levels is correlated to vitamin C, which indicates that all the antioxidants are closely interlinked [57].

Among the antioxidants, the three main enzymes CAT, GPx, and SOD are the first line of protection against oxidative stress [58]. SOD converts the superoxide anion to H_2_O_2_ and O_2_. Furthermore, H_2_O_2_ reduction is catalyzed by CAT and GPx, which guards the tissue against biomolecule-damaging ROS [59]. GST is a Phase 2 enzyme, which has a vital function in detoxification of the xenobiotics by converting it into more hydrophilic compounds in conjugation with GSH [60,61]. CCl_4_ triggered a marked reduction in the antioxidant enzymes SOD, CAT, and GPx and their levels were returned to near control values after EEAB treatment. The in vitro and in vivo antioxidant activity, including the free radical scavenging activity of EEAB, was reported earlier [10,25].

Alterations in gene expression after chronic toxicant exposure have a major influence on disease advancement [62]. Microarray studies have revealed that CCl_4_ causes gene expression changes, which provides a molecular response to CCl_4_ toxicity [63]. Our studies show that the gene expression levels of SOD, CAT, and GPx were down regulated after CCl_4_ treatment, and these results are in good accordance with other studies [16,64]. Depletion of antioxidant enzymes during CCl_4_ toxicity not only causes oxidative stress, but also causes impairment of enzymatic structure and function [16,65]. Furthermore, EEAB treatment considerably augmented the gene expression levels of all the studied antioxidants. Previous studies have revealed that polyphenols, such as hyperin, rotenone, and other plants extracts containing quercetin and rutin, significantly induce the gene expression of SOD, CAT, and GPx [16,64,66]. It has also been reported that the exogenous antioxidants during oxidative damage may promote the synthesis of antioxidant enzymes and, thereby, result in increased mRNA expression levels [67]. The present findings are in accordance with the earlier studies [16,67]. The alterations in isoform profiles (SOD1, CAT, GPx2, and GPx3) detected in the current study after oxidative stress induced by CCl_4_ can be related to alterations in the gene expression. These alterations in the isoforms pattern during stress have been ascribed to the changes in gene expression [68]. In this study, the discrepancy between gene expression and enzyme activity seen in the case of CCl_4_ induced stress may be due to the toxic effect of CCl_4_ being more pronounced at the post-transcriptional modification levels than at the post-translation modification levels. Furthermore, the complex regulation of gene expression mechanisms cannot be precisely associated with enzyme activity. However, the overall trend of antioxidant enzymes and gene expression during CCl_4_ stress conditions were in good agreement.

## 5. Conclusions

The present study demonstrates that EEAB confers protection against CCl_4_-induced oxidative stress mainly by augmenting the endogenous antioxidant levels and scavenging free radicals. Moreover, significant variations in isozyme profiles and down regulation of the gene expression of CAT, GPx, and SOD after CCl_4_ treatment were normalized after EEAB treatment. The phenolic and flavonoid compounds in EEAB identified using UHPLC-QTOF-MS may be responsible for its antioxidant and hepatoprotective activity. Additional studies are desirable on the active molecule isolation from EEAB to be used as a novel hepatoprotective agent.

## Figures and Tables

**Table 1:** Primer sequences used for the analysis of gene expression.

Gene Description	Primer Sequence (5′→3′)	Gene Bank Accession No.	Length (bp)
Cu-Zn-SOD	F: 5′GCAGAAGGCAAGCGGTGAAC	X05634	387
	R: 5′TAGCAGGACAGCAGATGAGT		
CAT	F: 5′GCGAATGGAGAGGCAGTGTAC	AH004967	670
	R:5′GAGTGACGTTGTCTTCATTAGCACTG		
GPx	F: 5′CTCTCCGCGGTGGCACAGT	M21210	290
	R: 5′CCACCACCGGGTCGGACATAC		
B-Actin *	F: 5′CTGCTTGCTGATCCACA	V01217	505
	R: 5′CTGACCGAGCGTGGCTAC		

* Housekeeping gene. F: Forward, R: Reverse.

**Table 2:** Phenolics and other compounds were identified in *Ammannia baccifera* ethanol extract by using UHPLC-QTOF-MS.

S. No.	RT (min)	Mass	Tentative Identification	Formula	DB Diff (ppm)
**Hydroxycinnamic Acids**					
1	4.37	354.0958	Chlorogenic acid	C_16_H_18_O_9_	−2.09
Flavonoids					
2	4.67; 6.65	448.0992	Quercetin	C_21_H_20_O_11_	2.96; 4.08
3	4.67; 6.43; 6.65; 9.16	304.0578	Pentahydroxy flavanone	C_15_H_12_O_7_	1.73; 2.58; 3.28; 3.29
4	6.05	432.1052	Cosmosiin	C_21_H_20_O_10_	1.1
5	6.02; 6.22;	320.0527	Dihydromyricetin	C_15_H_12_O_8_	1.75; 3.31
6	5.03	344.086	Eupatorin	C_18_H_16_O_7_	10.49
7	6.72	317.065	Petunidin	C_16_H_13_O_7_	3.71
8	0.82	138.0675	4-Hydroxyphenyl ethanol	C_8_H_10_O_2_	4.38
9	6.78	154.026	Protocatechuic acid	C_7_H_6_O_4_	3.65
Terpenoids					
10	4.56; 4.79	420.1618	Lamioside	C_18_H_28_O_11_	3.26; 1.41
11	14.7; 18.06	328.1665	Crocetin	C_20_H_24_O_4_	2.83; 2.38
12	4.07; 4.17	526.2536	Khayasin C	C_30_H_38_O_8_	5.75; 6.42
13	4.90	166.099	Perillic acid	C_10_H_14_O_2_	2.03
14	6.61	150.1038	(-)-Isopiperitenone	C_10_H_14_O	4.16
15	4.66	406.1458	10-Hydroxyloganin	C_17_H_26_O_11_	4.11
16	4.9	152.1197	(-)-trans-Carveol	C_10_H_16_O	2.88

**Table 3:** Effect of EEAB on lipid peroxidation (LPO) and lipid profiles in liver tissue of rats intoxicated with CCl_4_.

Parameter	Group I	Group II	Group III	Group IV	*p*-Value
LPO	1.83 ± 0.01 ^a^	3.94 ± 0.01 ^b^	3.08 ± 0.01 ^b,c^	2.15 ± 0.02 ^a^	0.041
Triacylglycerides	3.21 ± 0.01 ^a^	6.57 ± 0.05 ^b^	4.82 ± 0.04 ^c^	3.79 ± 0.02 ^a,c^	0.010
Cholesterol	3.10 ± 0.04 ^a^	4.22 ± 0.05 ^b^	4.02 ± 0.02 ^c^	3.72 ± 0.02 ^a^	0.010
Phospholipids	14.11 ± 0.03 ^a^	10.16 ± 0.05 ^b^	12.75 ± 0.16 ^c^	13.85± 0.04 ^a^	0.045

Treatments—I: control. II: CCl_4_ 30%. III: EEAB (250 mg/kg) + CCl_4_ 30%. IV: EEAB (500 mg/kg) + CCl_4_ 30%. LPO: nmoles of MDA/mg protein. Triacylglycerides, cholesterol, and phospholipids: mg/g tissue wt. Values are mean ± SEM (*n* = 3). Means with different suffix letters (a, b, c and d) differ significantly (*p* < 0.05).

**Table 4:** Effect of EEAB on protein damage (protein carbonyl and protein sulfhydryl) in liver tissue of rats intoxicated with CCl_4_.

Parameter	Group I	Group II	Group III	Group IV	*p*-Value
Total Protein	154.23 ± 0.54 ^a^	128.9 ± 0.32 ^b^	135.92 ± 0.45 ^c^	147.95 ± 0.39 ^a^	0.006
Protein Carbonyl	3.91 ± 0.01 ^c^	21.47 ± 0.17 ^a^	17.94 ± 0.15 ^d^	7.50 ± 0.22 ^a^	0.045
Total Sulfhydryl	3.10 ± 0.021 ^a^	0.91 ± 0.02 ^b^	1.53 ± 0.01 ^c^	2.87 ± 0.02 ^a^	0.030

Treatments—I: control. II: CCl_4_ 30%. III: EEAB (250 mg/kg) + CCl_4_ 30%. IV: EEAB (500 mg/kg) + CCl_4_ 30%. Total protein: mg/dL. Protein carbonyls: *n* moles/mg protein. Total sulfhydryl: mmoles/mg protein. Values are mean ± SEM (*n* = 3). Means with different suffix letters differ (a, b, c and d) significantly (*p* < 0.05).

**Table 5:** Effect of EEAB on enzymatic and non-enzymatic antioxidants in liver of rats intoxicated with CCl_4_.

Parameter	Group I	Group II	Group III	Group IV	*p*-Value
SOD	0.54 ± 0.01 ^a^	0.19 ± 0.00 ^b^	0.34 ± 0.00 ^c^	0.46 ± 0.00 ^d^	0.000
CAT	78.27 ± 0.57 ^a^	32.73 ± 0.16 ^b^	48.68 ± 0.16 ^c^	68.77 ± 0.27 ^d^	0.030
GPx	60.77 ± 0.80 ^a^	27.57 ± 0.64 ^b^	40.86 ± 0.28 ^c^	55.89 ± 0.29 ^c^	0.007
GSH	5.61 ± 0.15 ^a^	1.29 ± 0.02 ^b^	2.92 ± 0.02 ^c^	4.73 ± 0.15 ^d^	0.033
Vitamin C	3.10 ± 0.02 ^a^	2.14 ± 0.06 ^b^	2.56 ± 0.03 ^c^	2.81 ± 0.01 ^c^	0.014
Vitamin E	1.81 ± 0.01 ^a^	0.89 ± 0.02 ^b^	1.13 ± 0.03 ^c^	1.55 ± 0.02 ^d^	0.035

Treatments—I: control. II: CCl_4_ 30%. III: EEAB (250 mg/kg) + CCl_4_ 30%. IV: EEAB (500 mg/kg) + CCl_4_ 30%. CAT: µmoles of H_2_O_2_ utilized/min/mg protein. SOD: units/min/mg protein. GPX: µmoles of GSH oxidized/min/mg protein. GSH: µg of GSH/mg protein. Vitamin C & E: µg/mg protein. Values are mean ± SEM (*n* = 3). Means with different suffix letters (a, b, c and d) differ significantly (*p* < 0.05).

## Supplementary Materials

The following are available online at https://www.mdpi.com/1660-4601/16/8/1440/s1, Figure S1: Total chromatogram of Ammania baccifera ethanol extract in ESI positive mode by using UHPLC-QTOF MS.

## References

[1] Brautbar N., Williams J. (2002). Industrial solvents and liver toxicity: Risk assessment, risk factors and mechanisms. Int. J. Hyg. Environ. Health.

[2] Boll M., Weber L.W., Becker E., Stampfl A. (2001). Pathogenesis of carbon tetrachloride-induced hepatocyte injury bioactivation of CCI_4_ by cytochrome P450 and effects on lipid homeostasis. Z. Naturforsch. C.

[3] Hensley K., Robinson K.A., Gabbita S.P., Salsman S., Floyd R.A. (2000). Reactive oxygen species, cell signaling, and cell injury. Free Radic. Biol. Med..

[4] Kadri A., Zarai Z., Ben Chobba I., Bekir A., Gharsallah N., Damak M., Gdoura R. (2011). Chemical composition and antioxidant activity of *Marrubium vulgare* L. essential oil from Tunisia. Afr. J. Biotechnol..

[5] Lavanya G., Voravuthikunchai S.P., Towatana N.H. (2012). Acetone Extract from *Rhodomyrtus tomentosa*: A Potent Natural Antioxidant. Evid. Based Complementary Altern. Med..

[6] Modak M., Dixit P., Londhe J., Ghaskadbi S., Paul A., Devasagayam T. (2007). Indian Herbs and Herbal Drugs Used for the Treatment of Diabetes. J. Clin. Biochem. Nutr..

[7] Loganayaki N., Manian S. (2012). Antitumor activity of the methanolic extract of *Ammannia baccifera* L. against Dalton’s ascites lymphoma induced ascitic and solid tumors in mice. J. Ethnopharmacol..

[8] Loganayaki N., Siddhuraju P., Manian S. (2012). Antioxidant, anti-inflammatory and anti-nociceptive effects of *Ammannia baccifera* L. (Lythracceae), a folklore medicinal plant. J. Ethnopharmacol..

[9] Dash S., Das C., Sahoo D., Sahoo A., Nayak D. (2008). Preliminary phytochemical studies and antimicrobial activity of leaf of *Ammannia baccifera* (Linn.). Pharmacology.

[10] Lavanya G., Sivajyothi R., Parthasarathy P.R. (2008). In vitro Antioxidant Activity of Ethanolic Extract of *Ammannia baccifera* Linn. Bioscan.

[11] Montoro P., Maldini M., Russo M., Postorino S., Piacente S., Pizza C. (2011). Metabolic profiling of roots of liquorice (*Glycyrrhiza glabra*) from different geographical areas by ESI/MS/MS and determination of major metabolites by LC-ESI/MS and LC-ESI/MS/MS. J. Pharm. Biomed. Anal..

[12] Patti G.J., Yanes O., Siuzdak G. (2012). Innovation: Metabolomics: The apogee of the omics trilogy. Nat. Rev. Mol. Cell Biol..

[13] Zhang Q., Shi Y., Ma L., Yi X., Ruan J. (2014). Metabolomic Analysis Using Ultra-Performance Liquid Chromatography-Quadrupole-Time of Flight Mass Spectrometry (UPLC-Q-TOF MS) Uncovers the Effects of Light Intensity and Temperature under Shading Treatments on the Metabolites in Tea. PLoS ONE.

[14] Lavanya G., Manjunath M., Sivajyothi R., Parthasarathy P.R. (2010). Safety evaluation of the ethanol extract of *Ammannia baccifera* (Lythraceae): Assessment of Acute and Subacute toxicity. J. Pharm. Res..

[15] Luo Y.J., Yu J.P., Shi Z.H., Wang L. (2004). *Ginkgo biloba* extract reverses CCl_4_-induced liver fibrosis in rats. World J. Gastroenterol..

[16] Manubolu M., Goodla L., Ravilla S., Thanasekaran J., Dutta P., Malmlof K., Obulum V.R. (2014). Protective effect of *Actiniopteris radiata* (Sw.) Link. against CCl(4) induced oxidative stress in albino rats. J. Ethnopharmacol..

[17] Bradford M.M. (1976). A rapid and sensitive method for the quantitation of microgram quantities of protein utilizing the principle of protein-dye binding. Anal. Biochem..

[18] Ohkawa H., Ohishi N., Yagi K. (1979). Assay for lipid peroxides in animal tissues by thiobarbituric acid reaction. Anal. Biochem..

[19] Folch J., Lees M., Stanley G.H.S. (1957). A simple method for the isolation and purification of total lipides from animal tissues. J. Biol. Chem..

[20] Parekh A.C., Jung D.H. (1970). Cholesterol determination with ferric acetate-uranium acetate and sulfuric acid-ferrous sulfate reagents. Anal. Chem..

[21] Rice E.W., Roedrick P., McDonal R.P. (1970). Triglycerides in serum. Standards Methods in Clinical Chemistry.

[22] Van Handel E. (1961). Suggested modifications of the micro determination of triglycerides. Clin. Chem..

[23] Rouser G., Fkeischer S., Yamamoto A. (1970). Two dimensional then layer chromatographic separation of polar lipids and determination of phospholipids by phosphorus analysis of spots. Lipids.

[24] Levine R.L., Garland D., Oliver C.N., Amici A., Climent I., Lenz A.G., Ahn B.W., Shaltiel S., Stadtman E.R. (1990). Determination of carbonyl content in oxidatively modified proteins. Methods Enzymol..

[25] Lavanya G., Sivajyothi R., Manjunath M., Parthasarathy P. (2009). Fate of biomolecules during carbon tetrachloride induced oxidative stress and protective nature of *Ammannia baccifera* Linn.: A natural antioxidant. Int. J. Green Pharm..

[26] Misra H.P., Fridovich I. (1972). The Role of Superoxide Anion in the Autoxidation of Epinephrine and a Simple Assay for Superoxide Dismutase. J. Biol. Chem..

[27] Aebi H. (1984). Catalase *in vitro*. Methods Enzymol..

[28] Wendel A., Feuerstein S., Konz K.-H. (1979). Acute paracetamol intoxication of starved mice leads to lipid peroxidation in vivo. Biochem. Pharmacol..

[29] Moron M.S., Depierre J.W., Mannervik B. (1979). Levels of glutathione, glutathione reductase and glutathione S-transferase activities in rat lung and liver. Biochim. Biophys. Acta (BBA) Gen. Subj..

[30] Omaye S.T., Turnbull J.D., Sauberlich H.E. (1979). Selected methods for the determination of ascorbic acid in animal cells, tissues, and fluids. Methods Enzymol..

[31] Naziroglu M., Karaoglu A., Aksoy A.O. (2004). Selenium and high dose vitamin E administration protects cisplatin-induced oxidative damage to renal, liver and lens tissues in rats. Toxicology.

[32] Laemmli U.K. (1970). Cleavage of Structural Proteins during the Assembly of the Head of Bacteriophage T4. Nature.

[33] Chomczynski P., Sacchi N. (1987). Single-step method of RNA isolation by acid guanidinium thiocyanate-phenol-chloroform extraction. Anal. Biochem..

[34] Limaye P.V., Raghuram N., Sivakami S. (2003). Oxidative stress and gene expression of antioxidant enzymes in the renal cortex of streptozotocin-induced diabetic rats. Mol. Cell. Biochem..

[35] Weber L.W., Boll M., Stampfl A. (2003). Hepatotoxicity and mechanism of action of haloalkanes: Carbon tetrachloride as a toxicological model. Crit. Rev. Toxicol..

[36] Basu S. (2003). Carbon tetrachloride-induced lipid peroxidation: Eicosanoid formation and their regulation by antioxidant nutrients. Toxicology.

[37] Pathakoti K., Goodla L., Manubolu M., Tencomnao T. (2017). Metabolic Alterations and the Protective Effect of Punicalagin Against Glutamate-Induced Oxidative Toxicity in HT22 Cells. Neurotox. Res..

[38] Suman T.Y., Elumalai D., Kaleena P.K., Rajasree S.R.R. (2013). GC-MS analysis of bioactive components and synthesis of silver nanoparticle using *Ammannia baccifera* aerial extract and its larvicidal activity against malaria and filariasis vectors. Ind. Crops Prod..

[39] Michel T., Halabalaki M., Skaltsounis A.L. (2013). New concepts, experimental approaches, and dereplication strategies for the discovery of novel phytoestrogens from natural sources. Planta Med..

[40] Koolen H.H.F., da Silva F.M.A., Gozzo F.b.C., de Souza A.Q.L., de Souza A.D.L. (2013). Antioxidant, antimicrobial activities and characterization of phenolic compounds from buriti (*Mauritia flexuosa* L. f.) by UPLC-ESI-MS/MS. Food Res. Int..

[41] Choi H., You Y., Hwang K., Lee J., Chun J., Chung J.W., Shim S., Park C.-S., Jun W. (2011). Isolation and identification of compound from dropwort (*Oenanthe javanica*) with protective potential against oxidative stress in HepG2 cells. Food Sci. Biotechnol..

[42] Choi J.H., Kim D.W., Yun N., Choi J.S., Islam M.N., Kim Y.S., Lee S.M. (2011). Protective effects of hyperoside against carbon tetrachloride-induced liver damage in mice. J. Nat. Prod..

[43] Mikhaeil B.R., Badria F.A., Maatooq G.T., Amer M.M.A. (2004). Antioxidant and Immunomodulatory Constituents of Henna Leaves. Z. Nat. C.

[44] Kakkar S., Bais S. (2014). A review on protocatechuic Acid and its pharmacological potential. ISRN Pharmacol..

[45] Huang H.-C., Liao C.-C., Peng C.-C., Lim J.-M., Siao J.-H., Wei C.-M., Chen C.-C., Wu C.-S., Chang T.-M. (2016). Dihydromyricetin from *Ampelopsis grossedentata* inhibits melanogenesis through down-regulation of MAPK, PKA and PKC signaling pathways. Chem. Biol. Interact..

[46] Murray R.K., Granner D.K., Mayes P.A., Rodwell V.W. (1993). Harper’s Biochemistry.

[47] Lamb R.G., Snyder J.W., Coleman J.B., Testa B., Perrissaud D. (1988). New trends in the prevention of hepatocellular death. Modifiers of calcium movement and of membrane phospholipid metabolism. Liver Drugs: From Experimental Pharmacology to Therapeutic Application.

[48] Coleman J.B., Condie L.W., Lamb R.G. (1988). The influence of CCl_4_ biotransformation on the activation of rat liver phospholipase C in vitro. Toxicol. Appl. Pharmacol..

[49] Morrow J.D., Awad J.A., Boss H.J., Blair I.A., Roberts L.J. (1992). Non-cyclooxygenase-derived prostanoids (F2-isoprostanes) are formed in situ on phospholipids. Proc. Natl. Acad. Sci. USA.

[50] Wiggins D., Gibbons G.F. (1996). Origin of hepatic very-low-density lipoprotein triacylglycerol: The contribution of cellular phospholipid. Biochem. J..

[51] Kamalakkannan N., Rukkumani R., Viswanathan P., Rajasekharan K., Menon V.P. (2005). Effect of curcumin and its analogue on lipids in carbon tetrachloride-induced hepatotoxicity: A comparative study. Pharm. Biol..

[52] Dalle-Donne I., Rossi R., Giustarini D., Milzani A., Colombo R. (2003). Protein carbonyl groups as biomarkers of oxidative stress. Clin. Chim. Acta.

[53] Ritesh K.R., Suganya A., Dileepkumar H.V., Rajashekar Y., Shivanandappa T. (2015). A single acute hepatotoxic dose of CCl_4_ causes oxidative stress in the rat brain. Toxicol. Rep..

[54] Miesel R., Sanocka D., Kurpisz M., Kroger H. (1995). Antiinflammatory effects of NADPH oxidase inhibitors. Inflammation.

[55] Aquilano K., Baldelli S., Ciriolo M.R. (2014). Glutathione: New roles in redox signaling for an old antioxidant. Front. Pharmacol..

[56] Al-Dbass A.M., Al-Daihan S.K., Bhat R.S. (2012). Agaricus blazei Murill as an efficient hepatoprotective and antioxidant agent against CCl_4_-induced liver injury in rats. Saudi J. Biol. Sci..

[57] Winkler B.S. (1992). Unequivocal evidence in support of the nonenzymatic redox coupling between glutathione/glutathione disulfide and ascorbic acid/dehydroascorbic acid. Biochim. Biophys. Acta.

[58] Zhu R., Wang Y., Zhang L., Guo Q. (2012). Oxidative stress and liver disease. Hepatol. Res..

[59] Halliwell B., Gutteridge J.M. (2007). Free Radicals in Biology and Medicine.

[60] Habig W.H., Pabst M.J., Jakoby W.B. (1974). Glutathione S-transferases. The first enzymatic step in mercapturic acid formation. J. Biol. Chem..

[61] Mates J.M. (2000). Effects of antioxidant enzymes in the molecular control of reactive oxygen species toxicology. Toxicology.

[62] Jiang Y., Liu J., Waalkes M., Kang Y.J. (2004). Changes in the gene expression associated with carbon tetrachloride-induced liver fibrosis persist after cessation of dosing in mice. Toxicol. Sci..

[63] Harries H.M., Fletcher S.T., Duggan C.M., Baker V.A. (2001). The use of genomics technology to investigate gene expression changes in cultured human liver cells. Toxicol. In Vitr..

[64] El-Sayed Y.S., Lebda M.A., Hassinin M., Neoman S.A. (2015). Chicory (*Cichorium intybus* L.) root extract regulates the oxidative status and antioxidant gene transcripts in CCl4-induced hepatotoxicity. PLoS ONE.

[65] Szymonik-Lesiuk S., Czechowska G., Stryjecka-Zimmer M., Slomka M., Madro A., Celinski K., Wielosz M. (2003). Catalase, superoxide dismutase, and glutathione peroxidase activities in various rat tissues after carbon tetrachloride intoxication. J. Hepatobiliary Pancreat. Surg..

[66] Sanchez-Reus M.I., Gomez del Rio M.A., Iglesias I., Elorza M., Slowing K., Benedi J. (2007). Standardized *Hypericum perforatum* reduces oxidative stress and increases gene expressionof antioxidant enzymes on rotenone-exposed rats. Neuropharmacology.

[67] Ng T.B., Gao W., Li L., Niu S.M., Zhao L., Liu J., Shi L.S., Fu M., Liu F. (2005). Rose (*Rosa rugosa*)-flower extract increases the activities of antioxidant enzymes and their gene expression and reduces lipid peroxidation. Biochem. Cell Biol..

[68] El-baky A., Hanaa H., Hussein M. (2003). Influence of salinity on lipid peroxidation, antioxidant enzymes and electrophoretic patterns of protein and isoenzymes in leaves of some onion cultivars. Asian J. Plant Sci..

