# Supplementary material for: Protective Effects of Ammannia baccifera Against CCl4-Induced Oxidative Stress in Rats
Source: Int J Environ Res Public Health. 2019 Apr 23;16(8):1440. doi: 10.3390/ijerph16081440 (PMC6517918; doi:10.3390/ijerph16081440)
Supplement: Supplementary file 1 [file ijerph-16-01440-s001.pdf]

International Journal of
Environmental Research and Public Health

Supplementary Materials

Protective Effects of *Ammannia baccifera* Against CCl_4-Induced Oxidative Stress in Rats

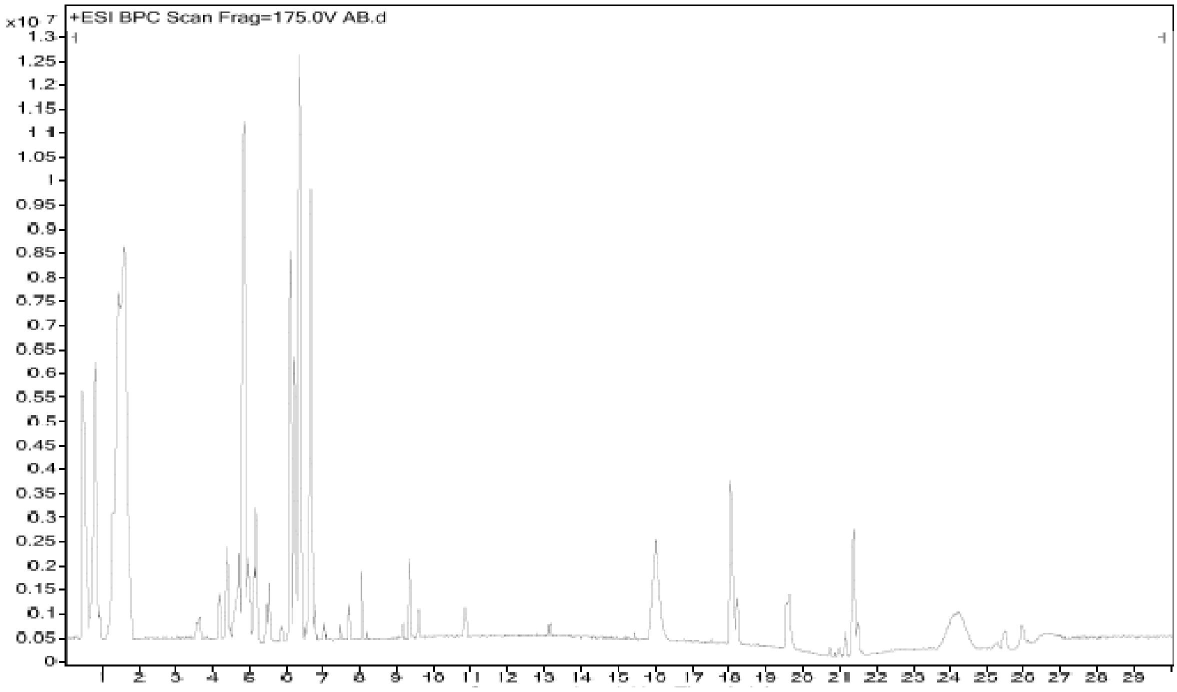

Figure S1. Total chromatogram of *Ammania baccifera* ethanol extract in ESI positive mode by using UHPLC-QTOF MS.
